# From dialogue to action: Assessing best practices and actionable steps for oral health professions education clinical and learning environments

**DOI:** 10.1002/jdd.13745

**Published:** 2025-05-27

**Authors:** Carlos S. Smith, Shelvia English, Ryan L. Quock, Steven Krzanowski

**Affiliations:** ^1^ Department of Dental Public Health and Policy VCU School of Dentistry Richmond Virginia USA; ^2^ Office of Access, Diversity and Inclusion American Dental Education Association Washington District of Columbia USA; ^3^ Department of Restorative Dentistry and Prosthodontics University of Texas School of Dentistry at Houston Houston Texas USA; ^4^ Equity, Diversity, Inclusion & Justice, Nonprofit HR Los Angeles California USA

**Keywords:** academic climate study, belonging, campus climate study, culture, cultural climate study, dental, diversity, education, equity, humanistic environment, inclusion, professionalism

## Abstract

The American Dental Education Association (ADEA) embarked upon a path to broadly assess the clinical and learning environments across dental school and allied oral health programs. Assessing clinical learning environments has provided an important opportunity for gaining feedback from learners and team members about their experiences, responding to their needs on an intentional basis and forming context‐specific understanding of and evidence for the impact of curriculum, pedagogical, and even patient care practices. This article presents key highlights of the overall usefulness of climate studies across higher education, corporate environments and health professions education. Moving from dialogue to action, it presents clear recommendations for moving forward and maximizing change efforts toward optimally inclusive, equitable, and ethically‐centered oral health professions education clinical and learning environments.

## 1 INTRODUCTION

It is important to understand how students, faculty, staff, and administrators experience the campus environment, which influences social interactions, work conditions, and learning outcomes for the campus community.^1^, ^2^ Since the 1990s, research on campus climate has centered around cross‐racial interactions and racial/ethnic composition related to affirmative action, given the historical and contemporary context of race within the United States.^3^, ^4^ In particular, researchers wanted to understand how students of color experience the college environment. Since then, campus climate studies have expanded to focus on more diverse, marginalized groups (e.g., LGBTQ+ and gender), emphasizing students’ experiences. Increasingly, faculty and staff perceptions are also captured. Commonly, climate study research focuses on undergraduate education, but increasingly graduate and professional schools and specific disciplines are seeing the value of climate studies for their programs and institutions.^1^, ^5^

The American Dental Education Association (ADEA) embarked upon a path to broadly assess the clinical and learning environments across dental school and allied oral health programs, which was no small task. A clear goal was furthering the important work of climate assessment for transforming and improving oral health education, which this special issue highlights. While the importance of clinical and learning environments that are diverse, equitable, inclusive, and cultivate belongingness is understood, more challenging has been the ability to measure the myriad forms of data that many climate studies have produced. The data, both quantitative and qualitative, is key. And even more important is the ability to correctly interpret it and move beyond mere sentiments of aspiration to implementation and actionable steps for change. Assessing clinical learning environments has provided an important opportunity for gaining feedback from learners and team members about their experiences, responding to their needs on an intentional basis, and forming a context‐specific understanding of and evidence for the impact of curriculum, pedagogical, and even patient care practices.^6^

## 2 WHY CLIMATE STUDIES

A primary benefit of climate surveys is the crucial first step of identifying differences in workplace and organizational culture between various groups.^7^ Campus climate assessments are intended to be used as an informative tool to help campus leaders and policymakers calibrate their institutions to change in a positive direction.

Some progress has been seen in dental education. A study by Ester et al. explored how many United States and Canadian dental schools participated in a climate study at their parent institution and/or conducted their own.^1^ The authors state:

“When asked which topics the climate studies at the parent institutions had assessed, the vast majority reported their academic units’ climate study assessed the climate overall as well as the climate for specific groups of community members. This result shows that the main focus of these studies was on the third CODA dimension of diversity, namely the institutions’ diversity‐related climate.^1^”

Climate assessments in oral health education specifically and the health professions broadly must continue to assess climate overall, and diversity, equity, inclusion (DEI), and belonging specifically. Yet, there is limited research demonstrating the implementation, impact, and success of climate assessment data on college and university climate, policies, and practices. As colleges and universities have made commitments and strides, DEI efforts still lag behind, especially in the wake of the recent decision to overturn the use of affirmative action in college admissions and the barrage of anti‐DEI legislation, universities backpedaling on commitments, and anti‐history claims. Administrators within health professions education must continue to move the needle beyond conducting assessments and make progress toward implementing changes based on assessment data to create substantive and sustainable changes across schools, programs, and the industry at large.

### 2.1 Climate studies at the college and university level

Across higher education, as well as amongst several health profession education institutions and industries, conducting climate studies has gained increased and continual prominence within the past decade. Climate studies may focus on varying issues including race and racism, LGBTQIA+ wellness, and wider issues of discrimination, harassment and incident reporting, and more. In a continually evolving understanding of stress, mental health, and improved well‐being, climate studies have shown significant positive correlations between a discriminatory campus climate and elevated levels of stress and anxiety, as well as a significant positive correlation between a discriminatory campus climate and depression.^8^ Time and time again historically marginalized faculty, including women of all racial and ethnic groups, experience the worst of campus environments.^9^, ^10^, ^11^, ^12^, ^13^

Climate studies can be useful for comparing the attitudes and actions of campus members across time and for comprehending the current conditions on campus. In fact, professors and administrators now have a better understanding of how different individuals on campuses may have drastically different perceptions and actions about the same spaces thanks to multi‐institutional climate studies and university‐specific climate assessments.^14^ Campus climate assessments are becoming a popular tool for academic institutions to use to learn more about what's going on on campus. As a result, these institutions need to be willing and ready to address how institutional structures, policies, and practices, as well as existing hierarchies, affect the perspectives and lived experiences of people on campus. However, many institutions find it difficult to turn their campus environment assessment results into meaningful changes.^15^

### 2.2 Climate studies within the workplace and industry

Oral health professions schools and programs have unique dual missions, they are educational institutions while also serving as clinical enterprises with revenue‐generating goals. Thus it is appropriate to understand how both higher education and corporate entities embrace and advance climate studies and their use. For example, corporate technology giant IBM has placed a priority on cultivating an optimal workplace and organizational climate. Their rationale: fostering a flexible and creative workplace yields innovation and job satisfaction. In fact, their internal studies showed a more than 25% variance in company productivity, innovation, and revenue dependent on team member morale and organizational climate.^16^ An organization's work environment fosters innovation, enhances employee perception, productivity, and effectiveness, and draws in and keeps talent. Key to their efforts were clear goals for improving all employee experiences, with a particular emphasis on addressing the workplace environment for women and historically marginalized groups, providing them with opportunities at work that are personally meaningful, and producing clear results where they are both valued and respected. Similar to best practices across industries and fields, IBM was clear in its commitments to diversity, which they define as inclusive of race, ethnicity, and gender, as well as the range of physical abilities, differences in culture, lifestyle, age, religion, economic status, sexual orientation, and marital status. Workplace diversity, according to IBM, is a “diversity house” built on three pillars: work/life balance initiatives, affirmative action, and equal opportunity.^16^ While affirmative action aims to remove disadvantages and give all groups a fair playing field on which to compete, equal opportunity is defined as the absence of discrimination and harassment. This aligns with both ADEA's and various industry best practices, not only as how to define diversity but also to underscore that climate studies must access all groups within an organization.^14^, ^17^

Other examples of corporate commitments include coffee titan Starbucks, which has had a myriad of public diversity and inclusion issues. Even still they provide key insights that climate studies and a commitment to principles of DEI, belonging, and access require a consistent and continual approach. To be clear, one‐and‐done Title IX or implicit bias training will not alone mitigate clinical and learning environments. In fact, Starbucks, and many other corporate and higher education institutions alike, have been clear in the rationale for long‐term strategic commitments. “Companies committed to creating a positive culture may well need to focus on trainings that help frontline staff become aware of their biases. This cannot be achieved in a few hours; it requires a conscious, long‐term investment.^18^”

Efforts in improving workplace climate and culture have yielded increased revenues and sales, a higher return on sales, and a decrease in staff turnover following said productivity. Building inclusive environments with evidence‐based procedures that prioritize environments and systems is essential. An organization that is really inclusive fosters a sense of value and belonging in its members, understanding that their contributions are essential to the organization's success. Establishing ecosystems based on diversity and equity requires first acknowledging and then tearing down exclusionary institutions and policies. Leadership must be deliberate, accountable, and responsive during this process. Furthermore, maintaining inclusivity depends on the function of corporate responsibility, which is achieved through monitoring performance indicators, evaluating employee engagement and satisfaction, and routinely reviewing established goals and objectives.

### 2.3 Climate studies across the health professions

Across what many consider to be helping professions, social work, medical education, nursing, and even veterinary medicine have encouraged the continual use of climate assessments. In fact, leading the charge with robust and continuing climate assessments has been the nursing and veterinary fields.^5^, ^19^ Academic nursing, spearheaded by the American Association of Colleges of Nursing (AACN) developed the Building a Culture of Belonging in Academic Nursing initiative in 2019, with particular emphasis on culture and belonging.^5^ As part of their efforts, AACN developed the Leading Across Multidimensional Perspectives (LAMP) Culture and Climate Survey. This survey assesses the nursing clinical and learning environments' impact on the cultural climate on student learning and outcomes. From their robust data collection and analysis processes across 3 years, they found that 85.8% of students, across racial/ethnic groups, had a strong sense of cohesion. Similar to findings in other climate studies, students of color were more likely to agree that subtle discrimination and microaggressions exist compared to white students. They also noted important findings for faculty and staff. The comprehensive report outlined several strategies for faculty and administrators particularly emphasizing the need for more inclusive teaching practices and to foster a team‐focused culture among faculty and staff to further aims of belongingness. Of the several recommendations for leaders, the report underscores leading by example, aligning policies and practices, and continuously assessing and adapting to improve culture and belonging for everyone. Leaders are tasked with carrying the mantle and burden of steering the direction of how culture is built and maintained.

The significance of leadership cannot be overstated. Leadership is a central necessity for moving climate studies from fact‐finding to true actionable change. Paramount for the entire landscape of oral health professions education to understand and grasp, is that leadership is far more than simply that which is tethered to position, title, or role. Leadership as a practice, an acquired skill, and a true competency to be attained must be both courageous and moral.^20^, ^21^ In fact, leadership absent of the centrality of DEI, belonging, well‐being, and access, is no true leadership at all. Intentionality would aim to prevent groups from being inherently left out of the equation or access altogether denied. Other innovations have seen academic health centers and health systems initiating the use of equity dashboards to evaluate the quality of treatment provided to particular patient population segments, and the techniques for utilizing this data are becoming more standardized and advanced. Many are also tracking how their employees feel about their organization's culture of inclusion, or the lack thereof. Regarding leadership, scholars note, “a simpler and more visible measure of diversity can be found in the photos of the leaders of health care organizations on their websites, and it is hard not to notice that they are mostly white and predominantly male.” In calling for diversity and inclusiveness amongst health care leadership, scholars posit three key actions: (1) recognize that diversity is necessary but does not, alone, ensure an inclusive and justice‐centered culture, (2) be aware that every leader is at risk for deficiencies and biases, and (3) appreciate that concepts of leadership and stereotypical traits of leaders among existing leaders may limit efforts for cultural inclusiveness and operational success.^22^ Could oral health professions adopt similar efforts? Is dental education finally ready to do things differently?

Veterinary medicine embarked upon a similar industry‐wide climate study in 2011. Their rationale centered on striving for admission goals that meet the needs of society at large.^19^ The goal was to investigate how a non‐supportive college climate linked to low diversity and poor inclusivity at veterinary institutions has been suggested as a major barrier to the recruitment and retention of people from various groups underrepresented in veterinary medicine. While overall impressions of climate were considered acceptable by many, similar to the ADEA findings and the larger body of research, different groups of individuals can experience the same environment very differently. Their climate study highlighted the fact that, although student comfort levels at veterinary schools were generally good, some subpopulations have different experiences; these groups include the LGBTQIA and various historically underrepresented racial and ethnic (HURE) populations. HURE students reported hearing racist remarks from their peers anywhere from infrequently to regularly, according to nearly one‐third of them. More than 20% of LGBTQIA students said they occasionally to frequently overheard remarks made by other students that were homophobic. Comments regarding sexual orientation and race were more common among students than among college officials and professors. Unfortunately, the study found that students are more likely than any other group on campus to have unfavorable experiences linked to diversity at the hands of their fellow students. Follow‐up studies continue to show the need for schools and colleges of veterinary medicine, professional organizations, and workplaces to target improvements to support LGBTQ+ students and professionals and the development of measures tailored to this population.^23^

A key takeaway from the veterinary medicine climate study was the need for a greater emphasis on the ways in which learners are professionalized into the field, also known as professional identity formation. Views regarding the quality of institutional support and the availability of faculty or staff mentors provided a foundation for further investigation at the veterinary college level regarding the ways in which students are preparing for careers. Their climate study provided evidence that students who feel supported and have access to mentors have a more positive experience in veterinary school, even though not all needs may be covered by the college or that every student will have a mentor from faculty or staff. To enhance the student experience and long‐term professional performance, opportunities to coach prospective mentors and veterinary students on inclusive behavior and professionalism that reflect institutional principles and mission are necessary strategic interventions.

Key actionable steps following veterinary medicine's climate studies included evolved accreditation standards as well as school interventions.^24^ Accreditation changes included approval of modifications allowing for the integration of diversity, equality, and inclusion throughout multiple standards as opposed to a single, diversity‐focused standard. Specific changes included “how to communicate with a diversity of clients with cultural humility, collaboration with a diverse workforce, supporting DEI awareness programs for students, disability or accessibility services for students, recruitment and admission practices, and others.^24^” A focus on community members, inclusive of learners, faculty, and staff, with both visible and nonvisible disabilities is paramount for continued student success in the professional curriculum and high‐stakes testing situations. Thus an emphasis on accommodations for learning, and the formation and resourced support of affinity groups can provide educational opportunities for students as extracurricular or co‐curricular events and initiatives. Providing an inclusive environment as the responsibility of everyone involved in veterinary medical education and the profession was also a key takeaway. This aligns with the greater need for evolving our understanding and praxis of professionalism, and emphasis on a true DEI, belonging, and access lens.

While academic medicine has yet to implement a medical education‐wide climate study, they do greatly encourage climate studies as an effective measure and tool. Specifically, the rationale surrounds the goals of producing a “physician and scientific workforce that advances high‐quality research and culturally competent care, academic medical centers must assess their capacity for diversity and inclusion and leverage opportunities for improvement.^25^” Assessing institutional capacity for building and sustaining inclusive climates begins with true assessment and understanding of community members' feelings of involvement and engagement. The creation of the Diversity Engagement Survey (DES) provided a means for quantifying the circumstances under which institutional culture promotes inclusivity and engagement.^25^ As a diagnostic tool, it helps organizations create a plan for accomplishing their diversity objectives by enabling them to evaluate their engagement and inclusion initiatives. The DES is used as a benchmarking tool to identify how institutions are doing in terms of engagement and inclusiveness. All things considered, the DES has helped academic medical centers evaluate and strengthen their institutional capacity to innovate and adapt in the midst of this period of change in all facets of academic medicine and health care.

Academic medicine also provides a clear path for oral health professions education to follow in addressing bias and discrimination in the health professions' learning environments. Five key recommendations include: “ create systems to identify and address bias and discrimination; make the reduction of bias and discrimination an institutional priority; ensure comprehensive curricula to reduce bias and discrimination; ensure critical diversity in the health professions; and create an institutional culture of respect, inclusion, and equity.^26^” Climate studies from various medical and other health professions schools have not only provided proof of attempts to meet particular accreditation requirements pertaining to the learning environment, but also may be utilized to address community member stress, workload in curriculum, and instructional strategies.^6^

### 2.4 Affecting political winds

The proverbial, and for some almost literal, elephant in the room remains the current US political realities and implications of limiting educational approaches to issues of DEI, belonging, and access. While at best, many of these political movements seeking to ban diversity initiatives and programs claim to counter discriminatory actions, much amounts to the dismantling of decades of work and advancement for historically marginalized groups. So how does one advance climate study data, and remain in compliance with local statutes and regulations? First, the authors advise unequivocally to maintain positive relationships with university and program counsel and proport no legal advice in this article. Secondly, one must gain a working knowledge of what is actually allowable versus implied.

In Texas, although state universities are no longer allowed to have a DEI office or assign an individual employee/third party to fulfill the job and responsibilities of said office, universities are allowed to support and demonstrate their work in supporting “first‐generation college students; low‐income students; or underserved student populations.^27^” Although race, “color,” ethnicity, gender, and sexual orientation make up a large portion of Texas's ban, support for members of those communities is not completely barred by the law. The broad categories of first‐generation college students, low‐income students, and “underserved student populations” encompass a diverse range of students, including women, students of color, LBTQIA+, and international students. Therefore, this prohibition does not mandate that these student groups can no longer receive any kind of support. The Texas restrictions do not apply to academic coursework so topics crucial to healthcare professions education such as social determinants of health should remain and be expanded upon across curricula. While mandatory DEI training is not permitted at state universities, training that has gone through specified vetting processes and that is related to complying with a relevant court order, state law, or federal law does not fall under the purview of the current ban; for example, a federally mandated anti‐discrimination training may remain mandatory.^28^

In Florida, coursework itself is under scrutiny with the passage of House Bill 7, which controls the content of lessons on racial issues that can be taught in K‐20 schools and punishes offenders harshly.^29^, ^30^ Most profoundly, scholars have brought attention to the seemingly intentional vagueness of some of these anti‐diversity laws and mandates.^29^, ^30^ “The text's ambiguity makes it likely that instructors and administrators will err on the side of caution.^29^” An example can be seen in the prompt removal of antiracism statements across varying departments at the University of Central Florida.^29^, ^30^ While some institutions may move to relabel office and unit names, emphasizing belongingness and wellness, reassignment still leaves the work to be done.

With the newness of these laws and efforts, more research is needed. However, initial findings do little to assuage much of what is feared. In a survey of LGBTQIA‐identifying faculty employed at public universities, preliminary evidence shows that the “laws will result in significantly fewer out LGBTQ+ faculty members, less course coverage of LGBTQ+ topics, and a reduction in academic research on LGBTQ+ issues. Unchecked, ultimately, this could mean that in states with some of the most difficult environments for LGBTQ+ people, there could be less research to address LGBTQ+ health and income disparities and inform public policies and a generation of students with less exposure to LGBTQ+ issues and faculty mentorship and support.^31^” These anti‐diversity laws and mandates, and even the 2023 rejection of race‐conscious admissions in colleges and universities by the Supreme Court of the United States, are most concerning given institutional responses that in aiming for compliance and caution, are in some cases amounting to complete destruction. And in some cases, prematurely, for example, private institution Duke University obliterated a decades‐long scholarship program for African American students, as a proactive measure, though under no legal constraints to do so.^32^ Oral health professions education must comply with the law, however, should there not also be a way forward that no longer confines the ethos and praxis of DEI, belonging and access to niches teams or officers, but broaden the scope of student success, academic affairs, clinical operations and affairs, faculty development, recruitment and retention of staff, and overall excellence to be inclusive of the ethos and praxis of such principles.^33^

Akin to what scholars have coined as the political determinants of health, it may be time for dental education to recognize the dire severity of the ways in which it should advocate not only for its own needs but for the oral health needs of the public at large. Those political determinants of health involve the systematic process of structuring relationships, distributing resources, and administering power, operating simultaneously in ways that mutually reinforce or influence one another to shape opportunities that either advance health equity or exacerbate health inequities.^34^ Three areas can be distinguished among political determinants of health: voting, government, and policy. The potential elimination of DEI, belonging, and access programming and initiatives, particularly with their strong link to increasing and maintaining workforce diversity may possibly only be halted with one clear political determinant of health, voting.^35^, ^36^

### 2.5 Call to action

Oral health professions clinical and learning environments are at a key tipping point, armed with climate study results that in some ways offer promise and in other ways offer caution. Broadly, there is a need for a comprehensive strategy for cultural overhaul. The inaugural ADEA Climate Study makes it clear, that while overall clinical and learning environments are relatively positive, many of our most historically marginalized and systematically oppressed community members experience an altogether different environment—particularly with a politically charged landscape in the United States falsely claiming that DEI as principles and initiatives make universities less welcoming. Oral health professions education must unequivocally further entrench its commitment to these principles, refusing to accommodate the false neutrality pushing such misinformation. The myth that inclusion is anything but actually further perpetuates and sustains inequality. While oral health professions education has long been mission‐minded, aspirational statements and strategic diversity plans are hollow without the policies and support systems that help all learners, staff, and faculty succeed and feel a sense of belonging.

### 2.6 Recommendations for actionable change

Below is the beginning of a list of actionable steps informed by the larger ADEA Climate Study generally, and the reviewed findings and research highlighted throughout this article and special issue to catalyze change efforts of oral health professions education clinical and learning environments.

Leaders within oral health education are poised to further the important work of climate assessment. Armed with campus‐level data and key findings from the ADEA Climate Study, leaders are tasked with moving the needle forward in ways that effectively translate into humanistic clinical and learning environments. This special issue details a fraction of key findings to elucidate the ways in which the data can inform strategic actions in which the data is not merely “sitting on the shelf” but is put into meaningful practice. To be clear, each clinical and learning environment is unique and must be specific in enhancing the measurement and interpretation of data on climate studies to effectively inform actionable steps for change, ensuring that quantitative and qualitative data are considered for that specific setting. To that end, the following recommendations are just the beginning of the actionable steps that can be taken within oral health professions education.

Continual assessment, inclusive of all campus groups including but not limited to gender and gender identity, race and ethnicity, sexual orientation, disabilities, citizenship and immigration status, veteran status, socioeconomic status, and more.

Improving organizational culture and climate occurs over time and several iterations.^37^ Diversity is both inclusive and distinct.^38^ Diversity efforts must continue to address issues of equity and representation and ensure success and accessibility for historically underrepresented racial and ethnic identity groups. Oral health professions education must also address issues pertaining to, among other things, immigration, gender identity, sexual orientation, and religion. After that, we have to advance in several ways. Even within an ever‐expanding inclusion understanding, the intersections of identities and diversity of identities for historically underrepresented people must always remain a priority. One must also consider the ways in which climate survey data may inadvertently lead to overlooking individualized experiences, particularly when a demographic group is so few that anonymity is a concern. Data also may not capture the experiences of all individuals, particularly those who may feel uncomfortable, afraid, or hesitant to express their accurate opinions. A need for continual assessment over and above surveys, to include focus groups and regular mechanisms for feedback is a must. “It would be easy, and convenient, to attempt to nullify the racialized experiences of some; however, individual‐level experiences of people at multiple marginalized intersections typically reflect social‐structural systems of power, privilege, and inequality.^39^” See bulleted list of recommendations in Table 1.

**TABLE 1:** Recommendations for actionable change.

Continual assessment, inclusive of all campus groups including but not limited to gender and gender identity, race and ethnicity, sexual orientation, disabilities, citizenship and immigration status, veteran status, socioeconomic status, and more. A commitment to, understanding and operating from an intersectionality framework for best unpacking climate study results and implementing change. Commitment to equity‐minded leadership, addressing and mitigating power dynamics to include exploring and implementing various models of shared leadership. Pursuing health justice and naming the various forms of oppression (racism, sexism, misogyny, homophobia, transphobia, ageism, etc.) operating in clinical learning environments with clear imperatives for actionable, continual, and evolving change. A commitment to belonging and wellness. A reimagined professionalism, calling for the evolution of historically biased and supposed norms of professionalism. Continually examine the ways in which oral health professions curricula perpetuate clinical learning environments that center racism, oppression, and other barriers to truly inclusive environments. Commitments to inclusive pedagogy, cultural humility, and culturally responsive practices. Accountability and mandatory development for those community members who exhibit chronic behavior contrary to the principles of equity, diversity, inclusion, belonging, well‐being, and access. Commit to doing something, and follow through.

#### 2.6.1 A commitment to, understanding and operating from an intersectionality framework for best‐unpacking climate study results and implementing change

Leaders must integrate intersectionality as a framework for understanding and applying climate study results where cross‐sections of the community are impacted in multiple ways, which can further necessary change that impacts larger groups of the community. Far from representing a simple addition of social identities such as race (e.g., Black) plus gender (e.g., woman), the intersectionality perspective, for example, asserts that race and gender constitute each other such that one identity alone (e.g., gender) cannot explain the unequal or disparate outcomes without the intersection of the other identity or identities.^39^ Thus intersectionality serves as an introductory framework for both understanding climate study results and implementing interventions for sustained change and optimal clinical learning environments.

#### 2.6.2 A commitment to equity‐minded leadership, addressing and mitigating power dynamics to include exploring and implementing various models of shared leadership

Leadership as a practice is absolutely essential and must be equity‐minded at all levels. Leadership, most often noted as the ability to influence others, systems, or outcomes, is inextricably linked to DEI, belonging, and access as values and requirements for innovation and an optimal healthcare workforce and patient outcomes. Leaders must ask themselves and others questions that center equity in their decision‐making processes. The performative diversity and inclusion rhetoric must be substituted for transformative efforts to promote true equity and justice. Oral health professions education and oral health generally must inquire unto itself, and truly ask the necessary questions^40^:

- “Diversity asks”, “Who's in the room?” Equity responds: “Who is trying to get in the room but can't? Whose presence in the room is under constant threat of erasure?”
- Inclusion asks, “Has everyone's ideas been heard?” Justice responds, “Whose ideas won't be taken as seriously because they aren't in the majority?”
- Diversity asks, “How many more of [pick any minoritized identity] groups do we have this year than last?” Equity responds, “What conditions have we created that maintain certain groups as the perpetual majority here?”
- Inclusion asks, “Is this environment safe for everyone to feel like they belong?” Justice challenges, “Whose safety is being sacrificed and minimized to allow others to be comfortable maintaining dehumanizing views?”
- Diversity asks, “Isn't it separatist to provide funding for safe spaces and separate student centers?” Equity answers, “What are people experiencing on campus that they don't feel safe when isolated and separated from others like themselves?^40^”

#### 2.6.3 Pursuing health justice and naming the various forms of oppression (racism, sexism, misogyny, homophobia, transphobia, ageism, etc.) operating in clinical learning environments with clear imperatives for actionable, continual, and evolving change

Health justice is distinctively a social ethic of care that reframes the relationship between health care, public health, and the social determinants of health, and names subordination as the root cause of health inequities.^41^ It is imperative to name the various forms of oppression (e.g., racism, sexism, misogyny, homophobia, transphobia, ageism, etc.). It is no longer beneficial to ignore that which shall not be named, and instead, name it and address it head‐on as it continues to operate in clinical environments and has ripple effects on patients, students, staff, faculty, and the larger community. In order to identify and eliminate the impact of racism, classism, and other types of social and cultural bias on the development and execution of policies ostensibly intended to reduce health disparities, health justice necessitates a self‐critical mindset. A dedication to collective action based on community involvement, empowerment, and participatory parity is also necessary for achieving health justice.

#### 2.6.4 A commitment to belonging and wellness

Belongingness, or sense of belonging, is perhaps the most comprehensive measure and attribute across climate studies. Scholars defined belongingness as “a basic human need, a fundamental right, and defined as a feeling that members (of a group) matter to each other and to the group, which is largely affirmed, reflected, and signaled in (and through) a constellation of institutional policies, programs, and practices that conspire, converge, and cooperate to clearly communicate: You belong here.^42^” Assessing and thus bettering campus and clinical environments is not only a matter of principled commitments but literal learner and team member safety and wellbeing. Wellbeing, across the learning and clinical environment teams must be centered for optimal patient outcomes.

#### 2.6.5 A reimagined professionalism, calling for the evolution of historically biased, and supposed norms of professionalism

New generations of learners and team members require a reimagining of professionalism to include social activism and societal concern for advancing health equity.^43^ Given the history of bias associated with some professionalism standards, particularly those standards, practices, and rules that have heavily emphasized appearance, grooming, hairstyles, or cultural attire that actually communicate preference, as opposed to professional behavior or action, oral health professions education, needs to address this reimagined understanding of professionalism. Racial and ethnic consciousness must be incorporated into the definition and application of professionalism, as well as professionalism norms and competencies. This is essential to maintaining oral health's reputation as a reliable profession and preserving the social contract in which it is ingrained.

#### 2.6.6 Continually examine the ways in which our oral health professions curricula perpetuate clinical learning environments that center racism, oppression, and other barriers to truly inclusive environments

Oral health professions curricula must center health equity, including but not limited to true understanding and application‐based knowledge of social determinants of health (SDOH). The root causes attributed to social determinants of health, which many scholars now estimate account for nearly 80% of health outcomes for some, must be immersed within both pre‐clinical and clinical education. To be clear, no external rotation or service learning opportunity should be absent of concrete understanding and competency of social determinants of health knowledge. Outreach projects, charity events, and the like should not set the standard for learner and community member SDOH knowledge and understanding.^44^

#### 2.6.7 Commitments to inclusive pedagogy, cultural humility, and culturally responsive practices

Inclusive pedagogy, cultural humility, and culturally responsive care each hinge on a lifelong commitment to learner knowledge, self‐evaluation, and self‐critique. Inclusive pedagogy also centers faculty commitment to avoid epistemic violence, or how health professions curricula and the information systems used to create them can be violent for students, especially those from groups that are systemically marginalized. Epistemic violence is the name given to the process of harm in these circumstances.^45^ In order to confront epistemic violence, it is imperative that educators acquire the ability to scrutinize medical knowledge systems and their capacity to perpetuate injustices and disparities. Over and above cultural competence, cultural humility goes beyond the concept of cultural competence to include the recognition of power dynamics and imbalances, a desire to fix those power imbalances, and to develop partnerships with people and groups who advocate for others Institutional accountability.^46^ Culturally responsive practices incorporate the beliefs, values, and behaviors of patients’ social and cultural backgrounds so health information is relevant to them. These practices strengthen relationships between staff & families.

#### 2.6.8 Accountability and mandatory development for those community members who exhibit chronic behavior contrary to the principles of DEI, belonging, well‐being, and access

Initiatives that only attract marginalized attendees and their allies tend to only increase awareness among that demographic—“preaching to the choir”—while leaving bad actors unaffected.^47^ Holding community members accountable who exercise chronically problematic behaviors that are counter to the humanistic aims of DEI, belonging, well‐being, and access is vital to cultivating a healthy community. Our learning and clinical environments are only as good as our worst actors. Thus skills building for engaging in crucial conversation is of dire importance. Practices such as bystander interventions, how‐to sessions on difficult conversations, as well as human resource departments that create restorative pathways for offenders are examples of strategic steps being taken. All too often, there is fear of retaliation from workplace bullies who wield much influence, often at the expense of marginalized team members who are overworked and overproduce to survive.^48^, ^49^

### 2.7 Commit to doing something and follow through

Passive approaches don't bring about necessary transformation and actually increase workplace stressors.^50^ Picking one or two impactful initiatives to focus on and actually implementing will bring about more tangible results than a non‐implemented, multi‐prong approach.^51^

## 3 CONCLUSION

To be clear, there is no excellence within healthcare at large, nor oral health professions education specifically, without inclusion. Even the commonly evolved moniker of inclusive excellence, while bearing positive intentions, is perhaps misleading. Achieving and cultivating inclusive learners, clinicians and environments is in no way an add‐on or nice‐to‐do. Any centering of excellence, a hallmark of professionalism, education, and healthcare delivery must be DEI, belonging, and access‐centered. Climate studies provide a much‐needed mechanism for the assessment, accountability, and implementation of these needed measures. Oral health professions education and delivery of care will only be at their best when the clinical and learning environments they occur in are at their best alike. Let us dare to reimagine what a new oral health education professional community looks, sounds, and feels like…and get to work.

## DISCLAIMER

This article is based on empirical research. The analysis and interpretations expressed in this piece are solely those of the authors and not intended to represent or reflect the position or viewpoints of the American Dental Education Association, its publisher, or any entities with whom the authors may or may not be affiliated or associated.

[Correction added on 27 May 2025, after first online publication: A disclaimer section has been added to this version.]
